# Polyautoimmunity manifest as inflammatory myopathy, uveitis, and progressive cutaneous depigmentation in a mixed breed dog: a case report

**DOI:** 10.1186/s12917-023-03764-4

**Published:** 2023-10-10

**Authors:** Mary Ann Lee, Sean E. Hulsebosch, Verena K. Affolter, Jonathan D. Dear, Marguerite F. Knipe, David J. Maggs, Bret A. Moore, Catherine A. Outerbridge, Sina Marsilio

**Affiliations:** 1 School of Veterinary Medicine, University of California, Davis, Davis, CA USA; 2 Department of Medicine and Epidemiology, University of California, Davis, 1 Garrod Drive, Davis, CA 95616 USA; 3 Department of Pathology, Microbiology and Immunology, University of California, Davis, Davis, CA USA; 4 Department of Surgical and Radiological Sciences, University of California, Davis, Davis, CA USA; 5 Department of Small Animal Clinical Sciences, University of Florida, Gainesville, FL USA

**Keywords:** Polyautoimmunity, Uveodermatological syndrome, Vogt-Koyanagi-Harada disease, Inflammatory myopathy, Generalized depigmentation, VKH-like syndrome

## Abstract

**Background:**

Polyautoimmunity is the expression of more than one autoimmune disease in a single patient. This report documents polyautoimmunity in a mixed breed dog with concurrent uveitis, cutaneous depigmentation, and inflammatory myopathy.

**Case presentation:**

A 1-year-old male neutered mixed breed dog was presented for progressive generalized leukotrichia and leukoderma, bilateral panuveitis, and masticatory muscle atrophy. The latter progressed to myositis of lingual, pharyngeal, and masticatory muscles confirmed by biopsy. Temporalis muscle was completely replaced by adipose and fibrous tissue, and necrotic myofibers with extensive infiltration of mononuclear cells indicated active myositis of lingual muscle. Skin biopsies showed severe melanin clumping in epidermis, hair follicles, and hair shafts, and perifollicular pigmentary incontinence. Uveitis, depigmentation, and myositis affecting the masticatory, pharyngeal, and tongue muscles were diagnosed based on clinical, histological, and laboratory findings.

**Conclusions:**

To the authors’ knowledge, this is the first report of concurrent uveitis, progressive cutaneous depigmentation, and inflammatory myopathy in a dog.

## Background

Inflammatory myopathies are a group of disorders characterized by inflammatory cell infiltration within muscles, and can be infectious or immune-mediated [1]. Major canine diseases classified as immune-mediated inflammatory myopathies include polymyositis, dermatomyositis, and masticatory muscle myositis (MMM). Polymyositis is a generalized immune-mediated inflammatory myopathy affecting the striated muscles [2]. Diagnostic criteria include clinical signs such as muscle weakness, exercise intolerance, stiff gait, lameness, and myalgia; elevated serum creatine kinase (CK) activity; abnormal electromyography; and histopathological features of myositis in the absence of underlying infectious or neoplastic diseases [3].

MMM is an inflammatory myopathy which focally affects the muscles of mastication (i.e., digastricus, temporalis, and masseter). In MMM, antibodies against type 2 M muscle fibers cause masticatory muscle inflammation and atrophy, resulting in pain and difficulty opening the jaws [3–5]. MHC class I and II are both expressed in the muscle membrane of dogs with polymyositis and MMM independent of inflammatory cell infiltration, and may play an important role in the pathogenesis of these conditions [6]. In addition, multiple genes associated with macrophage and dendritic cell functions are upregulated in both disease processes [7].

Uveodermatological syndrome (UDS) in dogs, also referred to as Vogt-Koyanagi-Harada-like syndrome (VKH), is an uncommon autoimmune syndrome targeting melanocytes or associated antigens with recognized breed predispositions in Akitas, Samoyeds, Malamutes, and Siberian Huskies [8–10]. Clinical signs can include bilateral granulomatous panuveitis, retinal detachment, and secondary glaucoma, as well as subsequent or concurrent development of skin lesions, characterized by leukoderma and leukotrichia with variable erythema due to a markedly granulomatous lichenoid inflammation with fine melanin dusting within lesional histiocytes [5]. Secondary erosion or ulceration of the skin, alopecia, and crusting can also develop [9].

Polyautoimmunity is a rarely reported syndrome in veterinary medicine, where a patient concurrently has more than one autoimmune disease [11]. Polyautoimmunity is more widely recognized to occur in human medicine, and better characterized with a suspected or confirmed genetic basis, as well as shared clinical signs, symptoms, and pathophysiology, or autoimmune tautology [12, 13].

This case report describes the clinical presentation, laboratory findings, and histologic examination of a mixed breed dog presenting with concurrent inflammatory myopathy, uveitis, and generalized depigmentation clinically resembling UDS.

## **Case presentation**

**Fig. 1:**
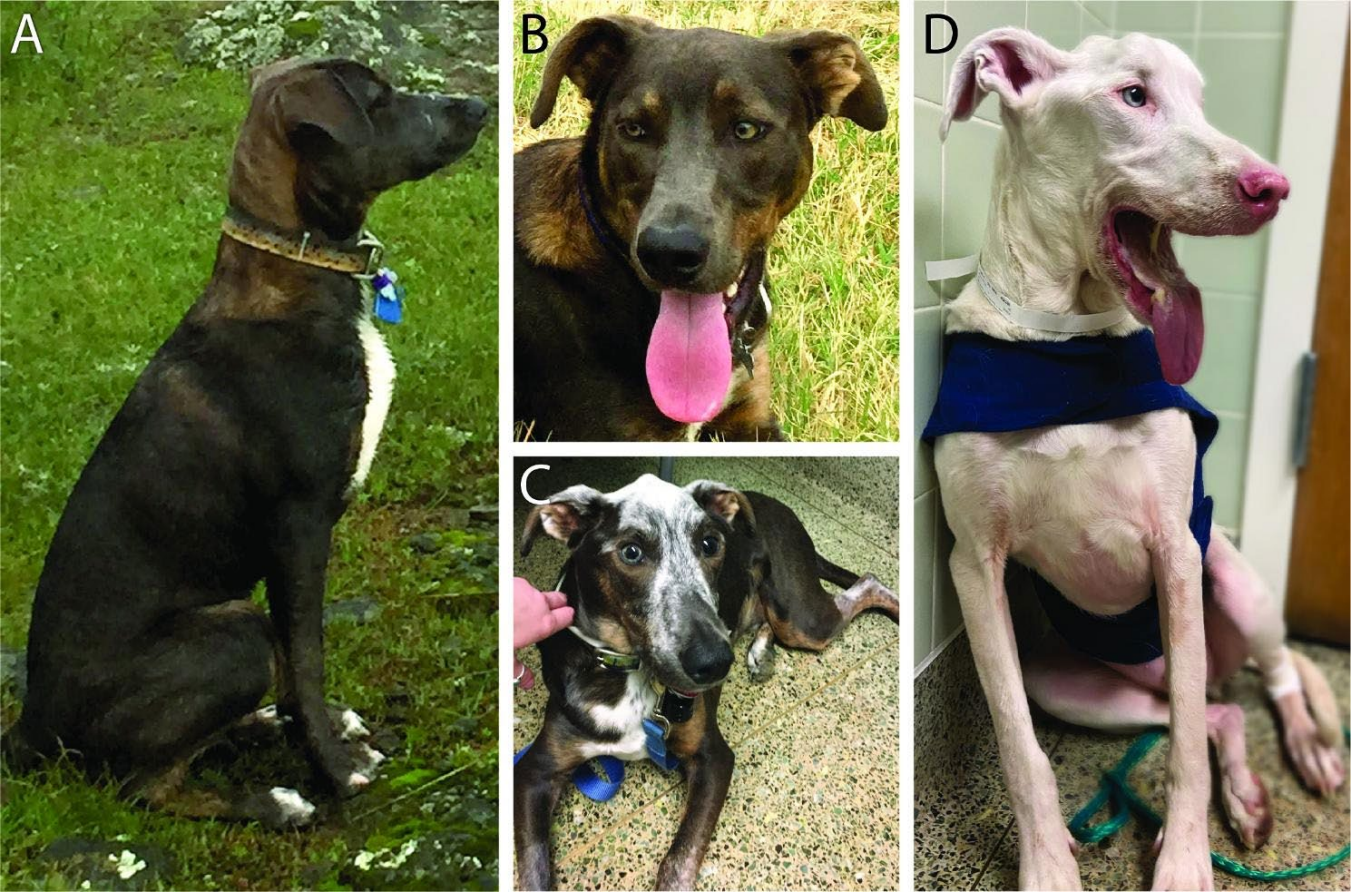
Photographs of a mixed breed dog with progressive cutaneous depigmentation associated with inflammatory myopathy and uveitis. **A&B.** Appearance at approximately 9 months of age, prior to onset of clinical signs. **C.** Appearance at approximately 1 year and 5 months of age (6 months after owner reported onset of cutaneous clinical signs). Note the bilateral masticatory muscle atrophy and leukotrichia of the head and ventral neck. **D.** Appearance at approximately 5 years of age (4 years and 1 month after owner reported onset of cutaneous clinical signs). Note the generalized leukotrichia and advanced atrophy of all muscles of mastication

A 1-year-old male neutered mixed breed dog presented to a Teaching Hospital Small Animal Internal Medicine Service with a history of unilateral uveitis, symmetrical atrophy of the temporal and masseter muscles, and leukotrichia and leukoderma of the top of the head. Four months before presentation, the dog developed partial alopecia involving the dorsal aspect of the head with subsequent development of leukotrichia in the affected area (Fig. 1A-C), as well as leukotrichia along all four distal limbs. Two weeks prior to presentation, the dog was seen by a veterinary ophthalmologist and diagnosed with chronic anterior uveitis OD and prescribed prednisolone acetate 1% drops (2 times daily OD, 4 times daily OS), meloxicam (dose not stated), and doxycycline (7.7 mg/kg PO once daily). The primary care veterinarian submitted serum samples for a combined ELISA test (4DX, IDEXX Laboratories, Inc., Westbrook, ME) for *Dirofilaria immitis* antigen, and *Borrelia burgdorferi*, *Ehrlichia canis*, *Ehrlichia ewingii*, *Anaplasma spp.* antibodies, which was negative.

On presentation to the teaching hospital, the dog was bright and alert with unremarkable vital parameters with moderate symmetric temporal and masseter muscle atrophy. Serum biochemistry panel and a complete blood count (CBC) revealed mildly elevated activities of alanine transaminase (ALT; 170; reference range 21–72 IU/L) and creatine kinase (CK; 530; reference range 55–257 IU/L), with no other abnormalities noted. Serum antibodies (IgG and IgM) against *Toxoplasma gondii* were not detected. An indirect fluorescent antibody test for *Neospora caninum* was low level positive at 1:40, and considered unlikely to reflect active infection. Serology for type-2 M muscle fiber antibodies was equivocal for masticatory muscle myositis (1:100, Neuromuscular Lab, University of California San Diego; <1:100 negative for MMM, 1:100 borderline antibody titer, > 1:100 consistent with MMM).

**Fig. 2:**
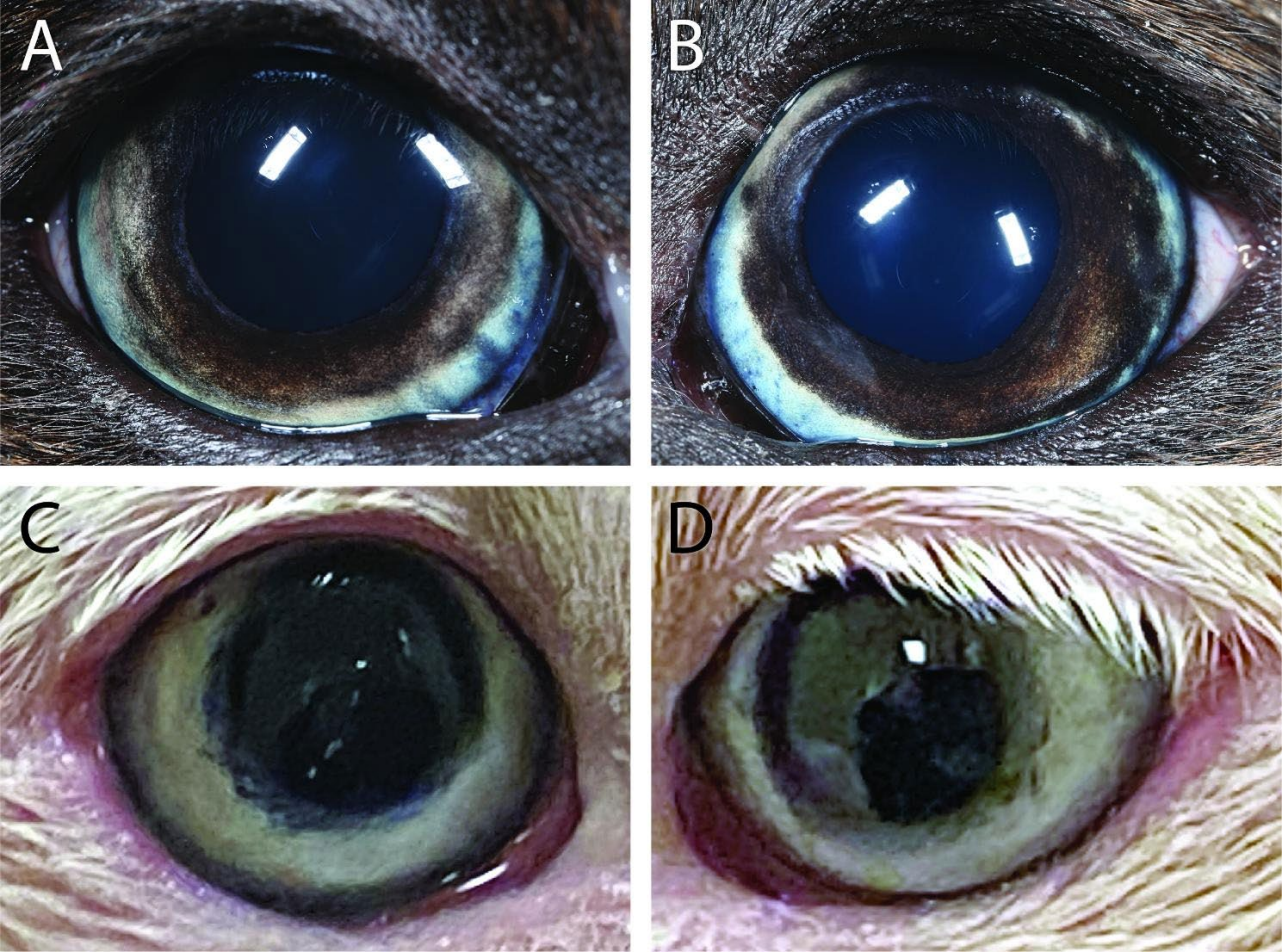
Photographs of the eyes of a mixed breed dog’s depigmentation of irides, skin, and uveitis. **A&B** Appearance at 1 year 3 months of age (1 month after owner reported onset of ocular clinical signs). **C&D** Appearance at 6 years and 2 months of age (5 years after owner reported onset of ocular clinical signs). Note the iridal color change in both eyes and marked depigmentation of the periocular skin and hair

A complete ophthalmic examination at the time of the next visit (2 weeks later) included slit lamp biomicroscopy and binocular indirect ophthalmoscopy before and after pupil dilation with 1% tropicamide. Both eyes were open and appeared comfortable, with normal globe position and movements OU. The dog was isocoric with brisk but incomplete direct and consensual pupillary light reflexes OU. Menace response, dazzle reflex, and palpebral reflex were normal OU, and the dog behaved as if visual in photopic conditions. The most striking abnormality was marked approximately sectoral heterochromia OU (Fig. 2A-B). In both eyes, there was a gradation of iridal melanosis from peripheral to axial with the iris root and ciliary zone being a steely blue-grey, the collarette more golden brown, the pupillary zone a dark brown-black, and the pupillary ruff a typical jet black. Dilated fundic examination revealed multifocal to coalescing areas of pigment absence throughout the non-tapetal fundus OU. Evidence of inflammation OD was limited to 1 + cell in the anterior chamber and 2 + pigmented cell in the vitreous, as well as posterior synechial remnants over the anterior lens capsule. In the left eye, the sole inflammatory finding was 1 + pigmented vitreous cell. There was no aqueous flare or conjunctival or episcleral hyperemia OU. The ophthalmic diagnosis was historic/controlled panuveitis OU consistent with uveodermatologic syndrome.

A neurological examination at the same visit revealed that the dog showed appropriate mentation and was ambulatory without overt paresis or ataxia. Cranial nerve examination revealed bilaterally absent maxillary lip pinch response, marked symmetrical atrophy of temporalis and masseter muscles, and slow but complete pupillary light reflex in both eyes, but was otherwise normal. Postural reactions and segmental reflexes were within normal limits. Palpation revealed appropriate range of motion in all four limbs with no atrophy of the limb muscles. The dog’s clinical signs were neuroanatomically localized to cranial nerve V (trigeminal nerve). Magnetic Resonance Imaging (MRI) pre- and post-contrast of the skull revealed marked muscle atrophy with T2 hypersensitivity and contrast enhancement of temporalis, masseter, and pterygoid muscles, suggestive of involvement of the masticatory muscles. MRI images also revealed mild to moderate bilateral lymphadenopathy of the mandibular and medial retropharyngeal lymph nodes. Cytological examination of a fine needle aspirate from the submandibular lymph node was consistent with reactive lymphoid hyperplasia. Evaluation of cerebral spinal fluid collected via cerebromedullary cisternal puncture was cytologically evaluated and assessed to be within normal limits.

Given the bilateral uveitis and ocular and cutaneous depigmentation, UDS was suspected, and Six-mm skin biopsies were collected from affected areas on the dorsal aspect of the head and the lateral aspect of the left pelvic limb at the level of the tibia. Histopathologic evaluation of hematoxylin- and eosin- (H&E-) stained tissue sections revealed severe melanin clumping within the epidermis, follicular epithelia, and hair shafts, as well as perifollicular and superficial melanin-laden macrophages (pigmentary incontinence) consistent with a dilute hair coat. There was no evidence of active inflammation in any of the biopsies or characteristic dermatopathologic pattern associated with UDS, despite the clinical suspicion. Systemic immunosuppressive therapy was initiated with prednisone at this point (2 mg/kg/day PO for 2 weeks, tapered to 1.5 mg/kg/day PO for 3 weeks, tapered to 1 mg/kg/day PO for 2 weeks).

Over a period of two months, no improvement in ocular or dermatologic signs was documented, and the clinical signs of masticatory muscle atrophy progressed. Corticosteroid therapy was discontinued after 8 weeks due to lack of clinical improvement, marked side effects, and the development of a cutaneous opportunistic fungal infection with *Scedosporium spp.* on the left thoracic limb after 1 week of corticosteroid treatment, which prompted treatment with voriconazole (10 mg/kg/day PO for 4 months). Lesions improved on voriconazole over a period of three months.

Dermatologic exam six months following presentation revealed progressive moderate to marked diffuse leukotrichia involving the face, chest, dorsum, and distal extremities (Fig. 1C). In addition, depigmentation of the mucocutaneous junctions and paw pads was noted. At this time, additional skin biopsies were collected due to development of subcutaneous-dermal nodules along the ventral neck, oral cavity, pinnal margins, and distal limbs. Six-mm biopsies from the ventral muzzle revealed severe, interstitial to diffuse, predominantly neutrophilic dermatitis admixed with lymphocytes, plasma cells, and mucin, as well as moderate periadnexal pigmentary incontinence and clumped melanin within follicles. Fungal culture and mycobacterium culture results were negative, and together with the biopsy results was suggestive of a secondary infectious process or sterile neutrophilic dermatitis.

Nine months post initial presentation to the teaching hospital, the dog developed progressive oral and pharyngeal dysphagia with inability to close the mouth and ptyalism. Thirteen months post presentation, a repeat type-2 M muscle fiber antibody was performed, and the results were negative (< 1:100, Neuromuscular Lab, University of California San Diego). A video fluoroscopic swallow study (performed sixteen months post presentation) using food admixed with barium (liquid barium, soft food, and kibble) revealed a gas-dilated pharynx, and documented base of the tongue and rostral pharyngeal weakness consistent with severe pharyngeal and lingual dysphagia. There was no evidence of gastroesophageal reflux or esophageal dysmotility.

An electromyogram (EMG) of major muscles of the head, limbs, and trunk was also performed on this visit, and revealed moderate to marked increased spontaneous activity in the temporalis, pharyngeal, lingual, and orbicularis oculi muscles, as well as increased insertional activity in the cranial tibial muscle. Muscle biopsies of the right temporalis muscle and the lingualis proprius muscle were also performed. Samples were oriented and flash-frozen in super-cooled butane, and stored at -80 degrees Fahrenheit for histochemical processing. Histopathological review of the temporalis muscle was consistent with an end-stage muscle, characterized by complete replacement of muscle fibers by adipose and fibrous tissue with no inflammatory cells present. Because of the end-stage changes in the temporalis, a primary disease process could not be determined (chronic denervation vs. chronic myopathy.) H&E-stained slides of the lingualis proprius muscle revealed a marked increase in connective tissue, small myofiber size, numerous necrotic myofibers, several central nuclei, extensive and multifocal cellular infiltration with macrophages and other mononuclear cells, consistent with severe, chronic myositis and end-stage scarring.

**Fig. 3:**
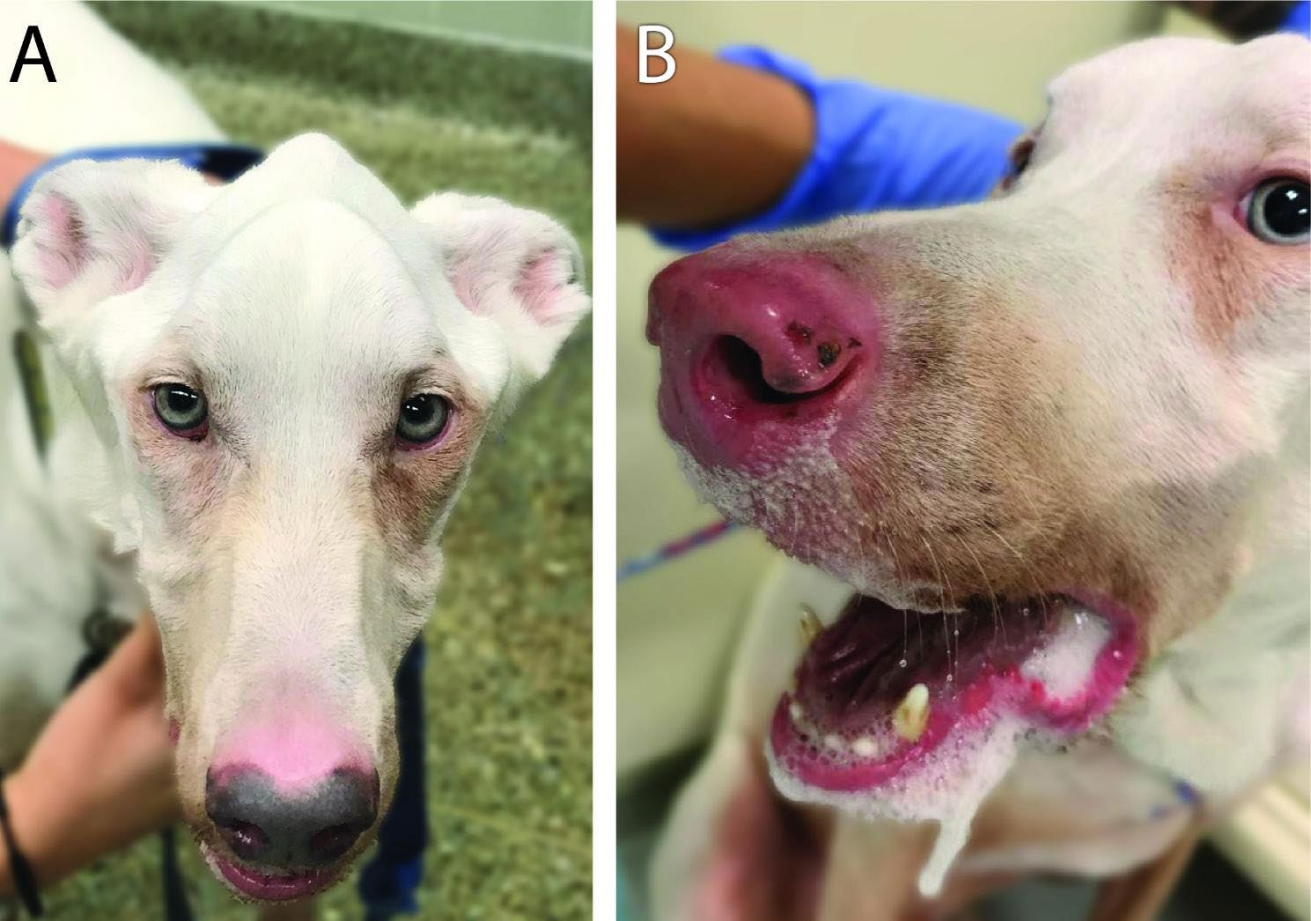
Photographs of a mixed breed dog with progressive depigmentation and inflammatory myopathy involving the masticatory muscles. **(A)** Appearance at approximately 3 years and 4 months of age (2 years and 5 months after owner reported onset of cutaneous clinical signs). Note marked iridal color change and severe masticatory muscle atrophy. **(B)** Appearance at approximately 4 years and 3 months of age (3 years and 4 months after owner reported onset of cutaneous clinical signs). Note depigmentation of the nose and oral mucocutaneous junctions, as well as iridal color change, marked ptyalism, and inability to close the mouth

Sixteen months following initial presentation, the dog had generalized leukotrichia and leukoderma, marked iridal depigmentation, generalized muscle atrophy including signs of temporomandibular disorder with jaw-closing weakness, and subsequent ptyalism (Fig. 3A-B). A repeat serum biochemistry revealed mildly elevated activities of ALT (104; reference range 21–72 IU/L), aspartate aminotransferase (AST; 83; reference range 20–49 IU/L), and CK (1189; reference range 55–257 IU/L). In light of negative tests for infectious diseases at presentation, and histopathological findings, progressive inflammatory myopathy involving masticatory, lingual, and pharyngeal muscles was suspected, and no further infectious disease testing was performed at this time. Treatment with cyclosporine (10 mg/kg/day PO for 3 months) followed by mycophenolate (25 mg/kg/day PO for the subsequent 2 months) did not lead to clinical or biochemical improvement. The dog’s dysphagia progressed, resulting in significant weight loss and prompting endoscopic placement of a percutaneous endoscopic gastrotomy (PEG) tube nineteen months after presentation.

Four years after initial presentation, the dog was maintaining stable body weight while being palliatively managed with nutritional needs and hydration provided via a PEG tube and cisapride (1 mg/kg/day through the PEG tube). Leukotrichia had progressed to involve the entire dog resulting in an entirely white hair coat and depigmentation of epidermis, as well as iridal depigmentation of both eyes (Fig. 1D C-D). A serum anti-nuclear antibody test was performed on this visit and found to be negative. Five and a half years following initial presentation, the dog succumbed to aspiration pneumonia and was euthanized due to grave prognosis. The owners declined necropsy.

## Discussion and conclusions

This case report describes a young, mixed breed male neutered dog initially presented with concurrent uveitis and depigmentation of hair coat and skin suggestive of UDS, as well as inflammatory myopathy involving the masticatory, lingual, and pharyngeal muscles. To the authors’ knowledge, there is one previous case report describing a similar case – a 1-year-old Jack Russell terrier presenting with polymyositis and signs of UDS, manifesting as uveitis and glaucoma with subsequent leukotrichia [14].

Coexistence of polymyositis and MMM with diagnostic 2 M antibody titers has been reported in 3 dogs and was referred to as “overlap syndrome” [15], a term used in humans with coexisting connective tissue diseases such as scleroderma and polymyositis [16]. At the original presentation, histopathologic findings in this dog were non-diagnostic for MMM despite clinically evident involvement of masticatory muscles, increased CK and ALT activities, and consistent MRI and EMG results. In addition, results of 2 M antibody tests for MMM were equivocal then negative with 2 separate test results. Corticosteroid administration may also have contributed to further atrophy of the masticatory muscles following initial presentation. Given the clinical progression with involvement of other muscle groups over time, the condition may be more adequately described as generalized inflammatory myopathy with involvement of masticatory muscles.

This dog’s ophthalmic signs included panuveitis, as well as iridal and chorioretinal depigmentation which, collectively, are highly suggestive of UDS. Unusually, this dog’s ocular inflammation remained reasonably well controlled with topical corticosteroids despite progressive uveal depigmentation. By contrast, most dogs with UDS experience progressive, bilateral panuveitis with anterior chamber pigment dispersion, iridal and chorioretinal depigmentation, retinal detachment, and often with sequelae such as glaucoma, posterior synechiae, cataracts, retinal atrophy, and optic nerve cupping or degeneration despite aggressive systemic immunosuppression [8].

The dermatologic lesions seen clinically in this dog included progressive bilateral symmetrical leukotrichia and leukoderma and mild erythema, starting on the head and progressing to a generalized pattern over the entire body, were also consistent with canine UDS [9]. However, dermatohistopathologic findings were not compatible with this diagnosis. The original skin biopsies revealed mild to moderate perifollicular pigmentary incontinence with associated pigment clumping, indicating a dilute hair coat with minimal perivascular inflammation. This is in contrast with histologic features expected with UDS, including marked lichenoid histiocytic and lymphoplasmacytic dermatitis with fine dusting of melanin in the cytoplasm of lesional histiocytes [17–19]. The previous report of the Jack Russell terrier with coexistent polymyositis and UDS was documented with classic histologic lesions of UDS [14]. The second set of skin biopsies in this dog also lacked diagnostic features of UDS.

It is possible that histologic and clinical diagnostic criteria of UDS were not both met in the current dog because he experienced an autoimmune disease similar but not identical to UDS, the selected biopsy site did not reflect diagnostic changes, or potentially because he was biopsied during a more quiescent or chronic disease stage with minimal inflammation [11]. The latter is often seen when a patient is biopsied while receiving immunosuppressive medications [18]; however, this dog was receiving only topical prednisolone acetate ophthalmic suspension at the time of skin biopsy collection. Although systemic absorption of prednisolone following ocular administration does occur, it is at only a fraction of the applied dose and below that typically required for treatment of dermatologic lesions in patients with UDS who require systemic immune-suppressive doses of prednisolone [9, 20]. Histopathologic lesions in human VKH syndrome can vary and thus diagnostic criteria in people mostly focus on clinical findings [17].

VKH syndrome, a condition similar to UDS in dogs, is the result of Th1 lymphocyte-mediated destruction of melanocytes through targeting of tyrosinase antigens with a strong association with leukocyte antigen haplotypes HLA-DRB1*0405 and HLA-DQB1*0401allele [9, 21]. Viral infections, including Epstein-Barr virus and cytomegalovirus, have been hypothesized as possible triggering factors for VKH syndrome in humans [21]. The importance of anti-retinal antibodies (ARAs) is still debated. One study reports a high association between the canine leukocyte MHC class II haplotype DLA-DQA1*00201 and the diagnosis of UDS in American akita dogs [9, 22] and a second report documents ARAs in an akita dog with UDS [23]. Other haplotypes of DLA class II have been associated with dermatomyositis (DRB1*015) and polymyositis (DRB1*02001/DQA1*00401/DQB1*01303) in Hungarian vizslas [24, 25]. Whether the clinical syndrome seen in the dog we describe had a genetic basis is unclear. No siblings or known relatives were located for this dog, and further investigation of the genetic basis of his polyautoimmunity was outside the scope of this case report.

A number of other immune-mediated diseases may be seen concurrently in in 30–45% of people with VKH syndrome [21]. These include Graves’ disease, IgA nephropathy, polyglandular autoimmune syndrome 1, Guillain-Barré syndrome, diabetes mellitus, ulcerative colitis, and multiple sclerosis [26–28]. Concurrent clinical signs reported in dogs in conjunction with UDS include lethargy, left head tilt, cranial nerve II deficit, change in behavior, hyporexia, transient pica, and depression [9]. However, whether these were directly associated with anti-melanocytic autoimmunity has been questioned, especially neurologic signs, since dogs have markedly fewer meningeal melanocytes compared to humans [29].

In this case, systemic immunosuppressive therapy with first-line (prednisone) and second-line (cyclosporine and mycophenolate) immunosuppressive drugs did not result in a notable clinical response. While the reasons for the lack of response are not clear, therapeutic failure following administration of immunosuppressive medications can occur in various immune-mediated diseases [30, 31]. In humans treated with glucocorticoids for inflammatory diseases, up to 30% of patients may experience treatment failures [32]. In addition, variable pharmacokinetics due to poor GI absorption and/or metabolism may have played a role.

In summary, this case report describes a 1-year-old male neutered mixed breed dog with panuveitis and inflammatory myopathy, ultimately affecting masticatory, lingual, and pharyngeal muscles, in conjunction with prominent leukotrichia and leukoderma, progressing over a span of almost 2 years. Coexistence of uveitis and progressive cutaneous depigmentation was highly suggestive of UDS, but skin biopsies examined lacked diagnostic histologic features. The ophthalmic clinical signs resolved with topical corticosteroid use leading to retention of vision; however, inflammatory myopathy and dermatologic signs progressed, and the dog was managed long term with alimentary supportive care. Polyautoimmunity, defined as two or more concurrent autoimmune diseases, is considered likely to have a genetic basis in humans [12, 13]. While it is not possible to pinpoint if a common pathophysiological mechanism was responsible for the varied clinical signs seen in this dog, polyautoimmunity appears to be an appropriate term to describe the combination of clinical signs documented in this case report. Clinicians should have increased clinical awareness of the possibility of the coexistence of multiple autoimmune diseases.

## Data Availability

All data used in the current study are available from the corresponding author on reasonable request.

## List of abbreviations

ALT: Alanine Transaminase
ARA: Anti-Retinal Antibodies
AST: Aspartate Aminotransferase
CBC: Complete Blood Count
CK: Creatine Kinase
EMG: Electromyogram
MMM: Masticatory Muscle Myositis
UDS: Uveodermatological Syndrome
VKH: Vogt-Koyanagi-Harada-like Syndrome

## References

[1] Pumarola M, Moore PF, Shelton GD (2004). Canine inflammatory myopathy: analysis of cellular infiltrates. Muscle Nerve.

[2] Kornegay JN, Gorgacz EJ, Dawe DL, Bowen JM, White NA, DeBuysscher EV (1980). Polymyositis in dogs. J Am Vet Med Assoc.

[3] Podell M (2002). Inflammatory myopathies. Vet Clin North Am Small Anim Pract.

[4] Shelton GD, Cardinet GH, Bandman E (1987). Canine masticatory muscle disorders: a study of 29 cases. Muscle Nerve.

[5] Shelton GD, Cardinet GH, Bandman E, Cuddon P (1985). Fiber type-specific autoantibodies in a dog with eosinophilic myositis. Muscle Nerve.

[6] Paciello O, Shelton GD, Papparella S (2007). Expression of major histocompatibility complex class I and class II antigens in canine masticatory muscle myositis. Neuromuscul Disord.

[7] Shelton GD, Hoffman EP, Ghimbovschi S, Peters IR, Day MJ, Mullins M (2006). Immunopathogenic pathways in canine inflammatory myopathies resemble human myositis. Vet Immunol Immunopathol.

[8] Zarfoss MK, Tusler CA, Kass PH, Montgomery K, Lim CC, Mowat F (2018). Clinical findings and outcomes for dogs with uveodermatologic syndrome. J Am Vet Med Assoc.

[9] Tham HL, Linder KE, Olivry T (2019). Autoimmune diseases affecting skin melanocytes in dogs, cats and horses: vitiligo and the uveodermatological syndrome: a comprehensive review. BMC Vet Res.

[10] Silpa-Archa S, Silpa-Archa N, Preble JM, Foster CS (2016). Vogt-Koyanagi-Harada syndrome: perspectives for immunogenetics, multimodal imaging, and therapeutic options. Autoimmun Rev.

[11] Anaya JM (2014). The diagnosis and clinical significance of polyautoimmunity. Autoimmun Rev.

[12] Anaya JM (2010). The autoimmune tautology. Arthritis Res Ther.

[13] Rojas-Villarraga A, Amaya-Amaya J, Rodriguez-Rodriguez A, Mantilla RD, Anaya JM (2012). Introducing polyautoimmunity: secondary autoimmune diseases no longer exist. Autoimmune Dis.

[14] Baiker K, Scurrell E, Wagner T, Walker D, Solano-Gallego L, Holt E (2011). Polymyositis following Vogt-Koyanagi-Harada-like syndrome in a Jack Russell terrier. J Comp Pathol.

[15] Evans J, Levesque D, Shelton GD (2004). Canine inflammatory myopathies: a clinicopathologic review of 200 cases. J Vet Intern Med.

[16] Alarcón-Segovia D (1994). Mixed connective tissue disease and overlap syndromes. Clin Dermatol.

[17] Herbort CP, Tugal-Tutkun I, Abu-El-Asrar A, Gupta A, Takeuchi M, Fardeau C (2022). Precise, simplified diagnostic criteria and optimised management of initial-onset Vogt-Koyanagi-Harada disease: an updated review. Eye (Lond).

[18] Gross TL, Ihrke PJ, Walder EJ, Affolter VK. Skin diseases of the dog and cat: clinical and histopathologic diagnosis. John Wiley & Sons; 2008.

[19] Kern TJ, Walton DK, Riis RC, Manning TO, Laratta LJ, Dziezyc J (1985). Uveitis associated with poliosis and vitiligo in six dogs. J Am Vet Med Assoc.

[20] Ewald MM, Rankin AJ, Meekins JM, Magnin G, KuKanich B (2022). Prednisolone and dexamethasone are systemically absorbed after topical application of ophthalmic suspensions in healthy dogs. Am J Vet Res.

[21] Lavezzo MM, Sakata VM, Morita C, Rodriguez EE, Abdallah SF, da Silva FT (2016). Vogt-Koyanagi-Harada disease: review of a rare autoimmune disease targeting antigens of melanocytes. Orphanet J Rare Dis.

[22] Angles JM, Famula TR, Pedersen NC (2005). Uveodermatologic (VKH-like) syndrome in american Akita dogs is associated with an increased frequency of DQA1*00201. Tissue Antigens.

[23] Murphy CJ, Bellhorn R, Thirkill C. Anti-retinal antibodies associated with vogt-koyanagi-harada-like syndrome in a dog. The Journal of the American Animal Hospital Association (USA); 1991.

[24] Hargis AM, Prieur DJ, Haupt KH, Collier LL, Evermann JF, Ladiges WC (1986). Postmortem findings in four litters of dogs with familial canine dermatomyositis. Am J Pathol.

[25] Massey J, Rothwell S, Rusbridge C, Tauro A, Addicott D, Chinoy H (2013). Association of an MHC class II haplotype with increased risk of polymyositis in hungarian Vizsla dogs. PLoS ONE.

[26] Mota LA, Santos AB. Vogt-Koyanagi-Harada’s syndrome and its multisystem involvement. Rev Assoc Med Bras (1992). 2010;56(5):590-5. 10.1590/s0104-42302010000500023 21152834

[27] Federman DG, Kravetz JD, Ruser CB, Judson PH, Kirsner RS (2004). Vogt-Koyanagi-Harada syndrome and ulcerative colitis. South Med J.

[28] Montero JA, Sanchis ME, Fernandez-Munoz M (2007). Vogt-Koyanagi-Harada syndrome in a case of multiple sclerosis. J Neuroophthalmol.

[29] Scott D, Miller W Jr, Griffin C. Immune-mediated disorders. *Muller & Kirk’s small animal dermatology*2001.

[30] Viviano KR, Glucocorticoids (2022). Cyclosporine, azathioprine, Chlorambucil, and Mycophenolate in Dogs and cats: clinical uses, Pharmacology, and Side Effects. Vet Clin North Am Small Anim Pract.

[31] Whitley NT, Day MJ (2011). Immunomodulatory drugs and their application to the management of canine immune-mediated disease. J Small Anim Pract.

[32] Creed TJ, Lee RW, Newcomb PV, di Mambro AJ, Raju M, Dayan CM (2009). The effects of cytokines on suppression of lymphocyte proliferation by dexamethasone. J Immunol.

